# Whales from Space: Counting Southern Right Whales by Satellite

**DOI:** 10.1371/journal.pone.0088655

**Published:** 2014-02-12

**Authors:** Peter T. Fretwell, Iain J. Staniland, Jaume Forcada

**Affiliations:** 1 Mapping and Geographic Information Centre, British Antarctic Survey, Cambridge, United Kingdom; 2 Ecosystems Department, British Antarctic Survey, Cambridge, United Kingdom; Natural History Museum of Denmark, Denmark

## Abstract

We describe a method of identifying and counting whales using very high resolution satellite imagery through the example of southern right whales breeding in part of the Golfo Nuevo, Península Valdés in Argentina. Southern right whales have been extensively hunted over the last 300 years and although numbers have recovered from near extinction in the early 20^th^ century, current populations are fragmented and are estimated at only a small fraction of pre-hunting total. Recent extreme right whale calf mortality events at Península Valdés, which constitutes the largest single population, have raised fresh concern for the future of the species. The WorldView2 satellite has a maximum 50 cm resolution and a water penetrating coastal band in the far-blue part of the spectrum that allows it to see deeper into the water column. Using an image covering 113 km^2^, we identified 55 probable whales and 23 other features that are possibly whales, with a further 13 objects that are only detected by the coastal band. Comparison of a number of classification techniques, to automatically detect whale-like objects, showed that a simple thresholding technique of the panchromatic and coastal band delivered the best results. This is the first successful study using satellite imagery to count whales; a pragmatic, transferable method using this rapidly advancing technology that has major implications for future surveys of cetacean populations.

## Introduction

“How many are there?” Is a question that is often difficult to address in ecology particularly for marine species that are generally inaccessible and cryptic. This is clearly demonstrated in whales where, despite their enormous size, robust population estimates are very difficult to obtain. The extreme size of whales means that they have a high per-capita rate of food consumption and hence a potentially massive impact on their prey populations as well as the marine ecosystem. Accurate population estimates are also essential to *inter alia* assess the recovery of depleted populations, evaluate conservation threats and also to use whales as indicators of the health of local ecosystems. Here we investigate the use of available Very High Resolution (VHR) satellite imagery to detect and count baleen whales as a proof-of-concept to augment current population studies. We target Southern right whales (*Eubalaena australis*) as a test species to evaluate; the southern right whale is an ideal subject for this work for many of the same reasons as it was an ideal whale to hunt, specifically its large size (maximum size ∼ 15 m) and a tendency, in the breeding season, to bask near the surface in large aggregations around sheltered coastal waters. This is particularly true for mothers that use shallow water areas to raise their calves to the surface during their first months of life. The techniques described in this paper may also be relevant to other species of baleen whales, especially other large whales that, like the southern right, breed in calm coastal waters. Further work to test availability and perception bias of counting whales by satellite will need to be completed before the techniques described here can be used to independently assess populations, such a system would reduce the observer cost and effort and improve the accuracy of population estimates and trajectories.

Southern right whales have a circumpolar distribution in the Southern Hemisphere. The distribution in winter, at least for breeding animals, is concentrated in shallow coastal waters in the northern part of their range [1]. In summer right whales are found mainly in latitudes 40–50°S [2] but have been seen, especially in recent years, in the Antarctic as far south as 65°S [3], [4] and around South Georgia [5], [6].

Southern right whales were hunted extensively from the 17^th^ through to the 20th century. The total number processed is conservatively estimated at about 155,000. The pre-whaling population was estimated at 55,000–70,000 dropping to a low of about 300 animals by the 1920s. After 1935 they were legally protected but over 3,000 more were thought to have been taken by illegal whaling in the 1960’s [7].

Since the cessation of whaling several southern right whale breeding populations (Argentina/Brazil, South Africa, and Australia) have shown a strong recovery [8], [9], [10] but the other breeding populations are still very small. In 1997 the estimated total population size was 7,500 animals and the three main populations have continued to increase [3], [11], [12]. Overall the population appears to have grown strongly since the cessation of whaling but is still at <15% of even conservative historical estimates.

Of current concern is the unprecedented mortality of southern right whales on their nursery grounds at Península Valdés, Argentina, in what are the most extreme mortality events ever observed in a baleen whale [13]. Over 420 whale deaths in recent years, the majority of which were calves, suggests that this population and its ecosystem may be less healthy and robust than previously thought [13].

The traditional methods by which cetacean population abundance estimates are obtained use counts of whales along transects from platforms such as aircraft or ships, or counts from land-based vantage points [14]. These can be very labour intensive involving long hours of recording by trained researchers and, as whales range over large geographic areas, these survey methods can be costly and inefficient. Additionally, not all individual whales are present at once, and if present they are not easily detectable (so called availability and perception bias, respectively). Detection probabilities for whales are typically high for shipboard surveys, but for the study area, where surveys are typically carried out by small airplanes, they can be down to 40% [15]. In addition, there are not many precision estimates for southern right whale abundance, particularly for the study area. Typically abundance is assessed with line transect methods and for right whales from the same population in the Scotia Sea coefficients of variation are wide, ranging from 65 to 185% [16].

A previous attempt to count whales using satellite remote sensing data and had limited success [17]. Using the first generation of VHR imagery from the Ikonos satellite, with a resolution of 0.8 m in the panchromatic and 3.3 m in the colour bands, two areas were looked at: the orca pools at SeaWorld theme park in San Diego, and a section of coastal water around Maui known to have large numbers of humpback whales [17]. Although objects which were probably whales were identified in the IKONOS imagery, the lower resolution and the cluster and noise associated with waves sea-surface state meant that definitive sightings were difficult to prove. Since 2002 the spectral, spatial and temporal accuracy of high resolution satellites has improved and cost of acquiring such imagery has decreased. A number of recent studies have used VHR satellites to count animals such as penguins and seals from space [18], [19], [20]. The highest accuracy satellite, the Worldview2 satellite, has an on-the-ground pixel size of 50 cm in the panchromatic and 2 m in its eight colour spectral bands. One of these bands, termed the coastal band, uses the far blue part of the spectrum to penetrate the water column and is routinely used for hydrographic mapping [21].

Here we describe a method of identifying and counting southern right whales breeding in part of the Golfo Nuevo in Argentina using satellite imagery from the WorldView2 satellite count. This is an ideal location to evaluate our methods because every year, from July to November, whales concentrate in high densities to calve and mate. These enclosed bays are characterized by calm and shallow waters increasing the chances of obtaining images with optimum conditions of visibility.

## Materials and Methods

**Figure 1:**
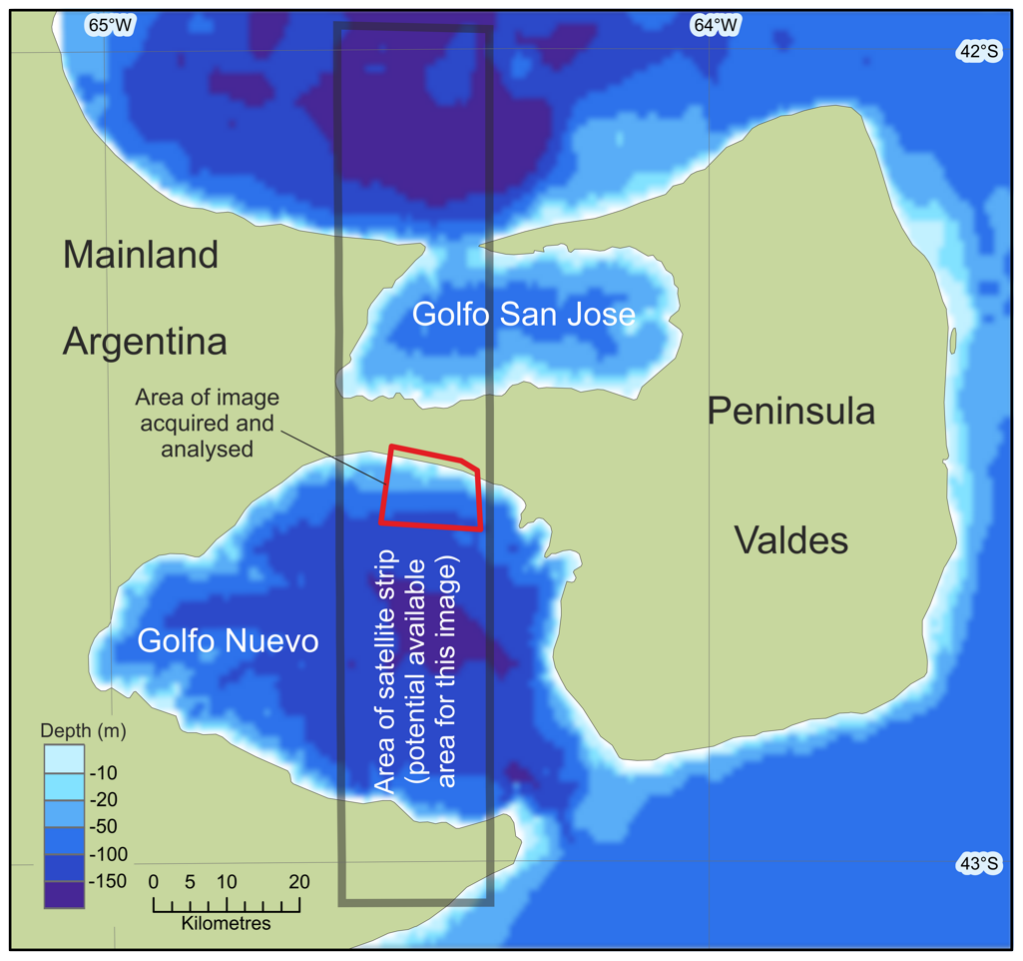
The area of the study and the location of places named in the text. The red box denotes the area of imagery acquired for this study. The grey area gives an indication of the possible swath width of a single satellite pass.

We acquired a single WorldView2 satellite image of a region of the Golfo Nuevo Bay, the southern of two bays which separate Península Valdés from the mainland of Argentina (figure 1).

### The location

Golfo Nuevo, the southern gulf of the Península Valdés, is a roughly circular shaped bay and between 80 – 100 km wide. The sheltered waters attract southern right whales in great numbers and, together with a similar sized bay just to the north, they hold one of the world’s largest breeding aggregations of the species. This represents one of the best studied populations of southern right whales, with an ongoing programme detailing the natural history and ecology of the species [5]. From July to November (winter and early spring), much of the population is on the nursery ground at Península Valdés [21] (42°S, 64°W). In 1997, the population was estimated at 2,577 whales [22], with an annual growth rate of 6.9% per year [10]. Current estimates are unavailable and are required as whale calf mortality has increased sharply since 2005 when the population has experienced several severe mortality events, particularly in Golfo Nuevo [13].

### The image

A section of a single WorldView2 image (Catalog ID: 103001001C8C0300) covering an area of 113 km^2^ and taken on the 19^th^ of September 2012 was purchased from the commercial provider Digital globe. The image was chosen from the Digital Globe archive for three reasons:

1. It covers the middle of the Golfo Nuevo Bay, an area with a high density of southern right whales.
2. The timing corresponds with the middle of the breeding/calf rearing season, which lasts between July and November.
3. It is cloud free with a calm sea-state.

Sea surface waves have a very strong influence on the ability to detect submarine features [17] Our previous analyses using VHR imagery in the Southern Ocean show that choppy water or sea swell refracts the sunlight making practical detection of whales almost impossible. This is especially true if attempting to construct routines to automatically identify targets. When choosing imagery from archival footage an online reduced resolution library is usually viewed. These reduced resolution “quick-looks” do not allow judgement on the sea-state, as they are too coarse to show surface waves – although whitecaps and swell lines can occasionally be seen. However several key features can indicate suitable calm conditions; these include sediment patterns and algal blooms and lack of surf at the coast.

The image acquired consists of nine bands of information; eight colour bands with an on the ground resolution of ∼2 m per pixel, (Digital globe http://www.digitalglobe.com/downloads/WorldView2-DS-WV2-Web.pdf) and one panchromatic band with an on the ground resolution of 50 cm. The fifth of the eight bands is termed the coastal band and collects light of wavelengths between 400 nm and 450 nm. This far-blue or violet light penetrates deeper into the water column with less absorption and attenuation than longer wavelengths (dependent upon water clarity and turbidity). This data is routinely used by hydrographic institutions for mapping coastal bathymetry [23].

**Figure 2:**
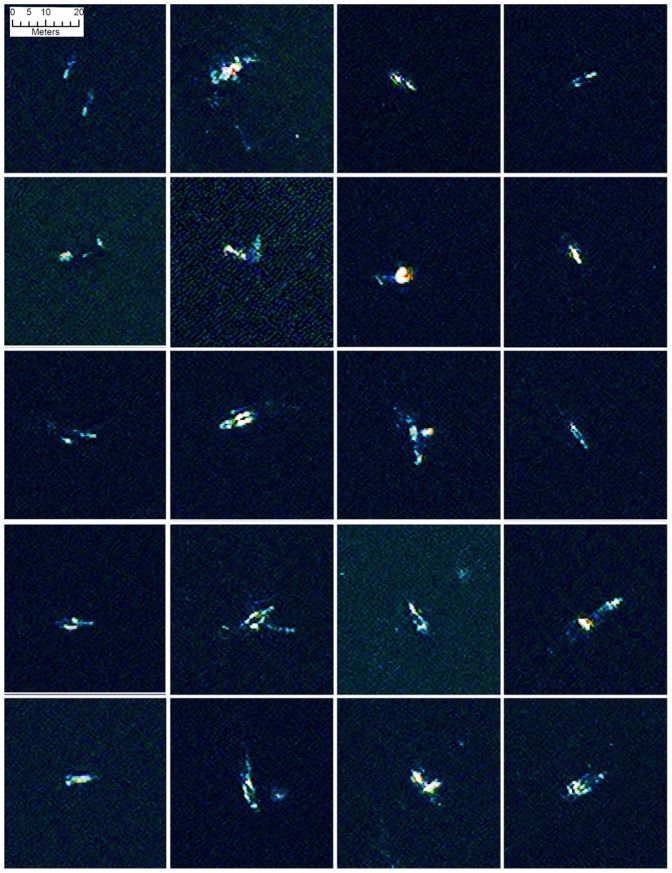
A selection of 20 comparable false colour image chips (bands 1-8-5) of probable whales found by the automated analysis. Several of the images could be interpreted as whale pairs, or as a mother and calf, others may be displaying behaviour such as tail slapping, rolling or blowing. On several images there is a strong return at one end of the feature which is mostly likely the calluses on the whales head. Reprinted under a CC BY license with permission from British Antarctic Survey and DigitalGlobe.

**Figure 3:**
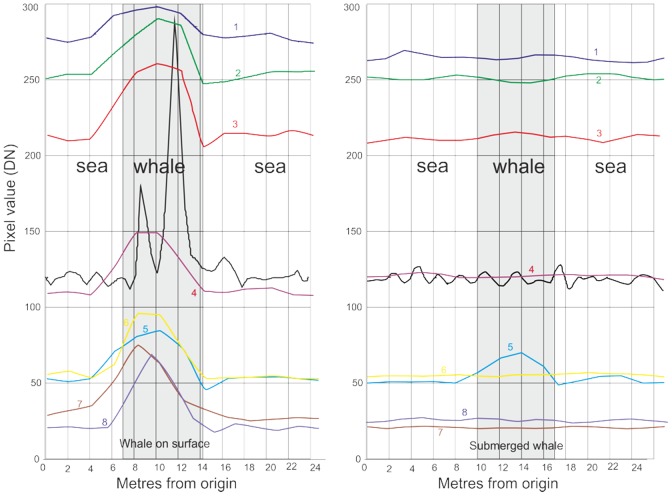
The sensor response through cross sections through two whale-like (correct shape and size) features assumed to be whales. The left hand figure is from a feature at the surface, the right hand figure shows a submerged feature. Note that while all bands show the surface feature, only band 5 (the Coastal Band) identifies the submerged feature.

We assessed the returns of each band over a cross section of pixels through whale-like features of two types; surface features and assumed submarine features (figure 2). As can be seen in figure 3, all bands responded to surface features, with the strongest response in the panchromatic band, although this band also showed the most noise. In the submerged cross section only the coastal band (band 5) responded, no other band showed evidence of any feature. This also shows the noisy nature of the panchromatic data in an area of open water (figure 3).

Previous attempts to identify whales using IKONOS imagery show that attenuation of light through the atmosphere is weak in comparison to the two major components of image degradation; scattering from surface roughness of the sea and attenuation of light through the water column due to water turbidity [17]. As these major components could not be quantified to absolute reflectance, absolute values for the subsurface features could not be retrieved, we therefore used raw Digital Number (DN) values from the satellite to give an indication of relative illumination across the image.

### Automatic detection

Using ENVI5 image processing software and ArcGIS automatic detection of whale-like features in the water column was tested using maximum likelihood supervised classification, unsupervised classification (isoData and k-means) and thresholding of specific bands.

Supervised classifications need the signatures input information of the pixel values for each class in order to classify the image. These signatures are usually manually input by the user. The algorithm then segregates all the pixels in the image into classes representing the signatures.

Unsupervised classifications classify the image into component parts based solely on information held within the image; isoData uses a clustering algorithm to determine the natural grouping of cells, while k-means calculates initial class means evenly distributed in the data space, then iteratively clusters the pixels into the nearest class using a minimum-distance technique.

Histogram thresholding [24] requires a degree of experimentation to calculate the best thresholds to use. Through an iterative process we formulated thresholds that maximized signal (in this case suspected whales), and reduced the amount of noise or false positives (single pixels from small objects and mixed pixels). As whales are large features they should be represented by multiple bright pixels, noise will result in single pixels, although inevitably there could be a small number of whales at depths that return only single pixels, so that some valid single pixels should still occur. Using the histograms of whale DN values as a guide we built thresholds that maximized the ratio of multiple pixels to single pixels in the panchromatic and coastal bands.

To construct a test dataset the image was divided up into a grid and whale-like features were manually digitized and coded into three classes: probable whale (features that were whale-shape and whale-sized) possible whale (including weaker signals, bubble slicks and some groups of seabirds are classed as possible whales*)*, or features only visible in Band 5 (The third class are objects identified only in the water penetrating coastal band, are interpreted as sub-surface feature that are also potentially whales). This process was conducted multiple times to ensure the lowest possible errors of omission.

## Results

**Figure 4:**
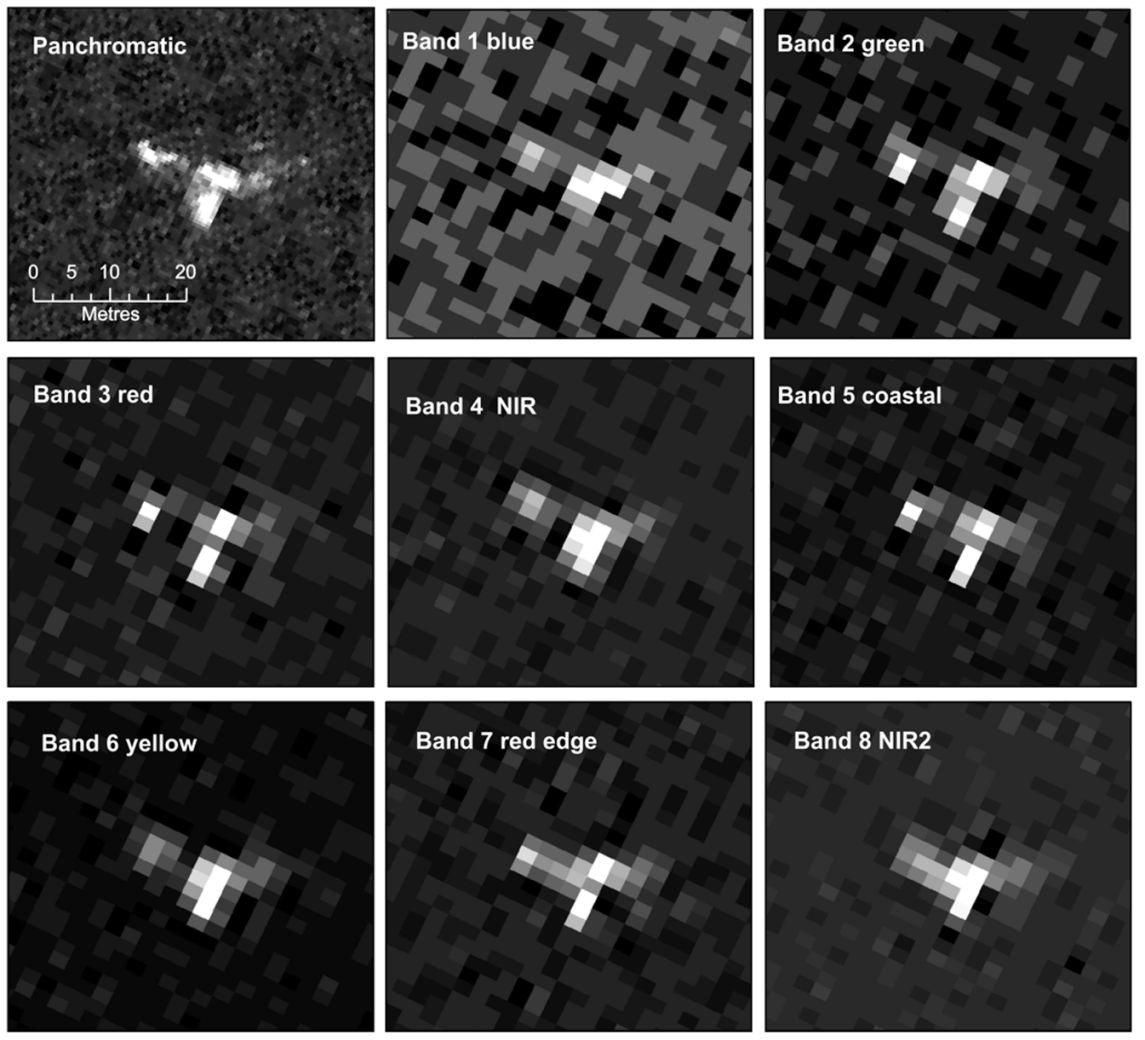
A single band images of a probable right whale in the satellite image from each of the eight multispectral bands and the panchromatic band of the WorldView2 data. Note that the higher resolution of the panchromatic band gives more detail, but it this increased detail also renders the object into several parts. Other bands show less detail, but have the advantage of homogenizing the object into one group of pixels, an important consideration when attempting to build automatic identification routines. Reprinted under a CC BY license with permission from British Antarctic Survey and DigitalGlobe.

Visual inspection of the image showed that a number of offshore objects, that were both the right shape and size (5 – 15 m) to be whales, could be identified in both the colour and the panchromatic bands (see figure 2). Most of these objects were visible across all bands although in most cases the high resolution of the panchromatic band rendered the objects in greater detail (figure 4). Visual inspection can only utilize three bands as the red, blue and green element of the onscreen image, we compared combinations of pansharpened bands to find the band-combinations in which whales were most visible, the best overall results were retrieved using a combination of bands 1 (red),8 (NIR2) and 5 (coastal). The panchromatic band alone displayed a higher noise ratio that other bands; possibly a result of the higher resolution picking up more surface refraction from wavelets, ripples and small waves. Other surface features such as aggregations of seabirds were also visible in this band. These smaller features were a confusing element when attempting to develop automatic recognition algorithms. The coastal band (band 5) identified a number of features not apparent in the other data that were interpreted as sub-surface features. Manual counts of the gridded dataset found 55 probable whales, 23 possible whales and 13 objects identified in Band 5 only.

**Figure 5:**
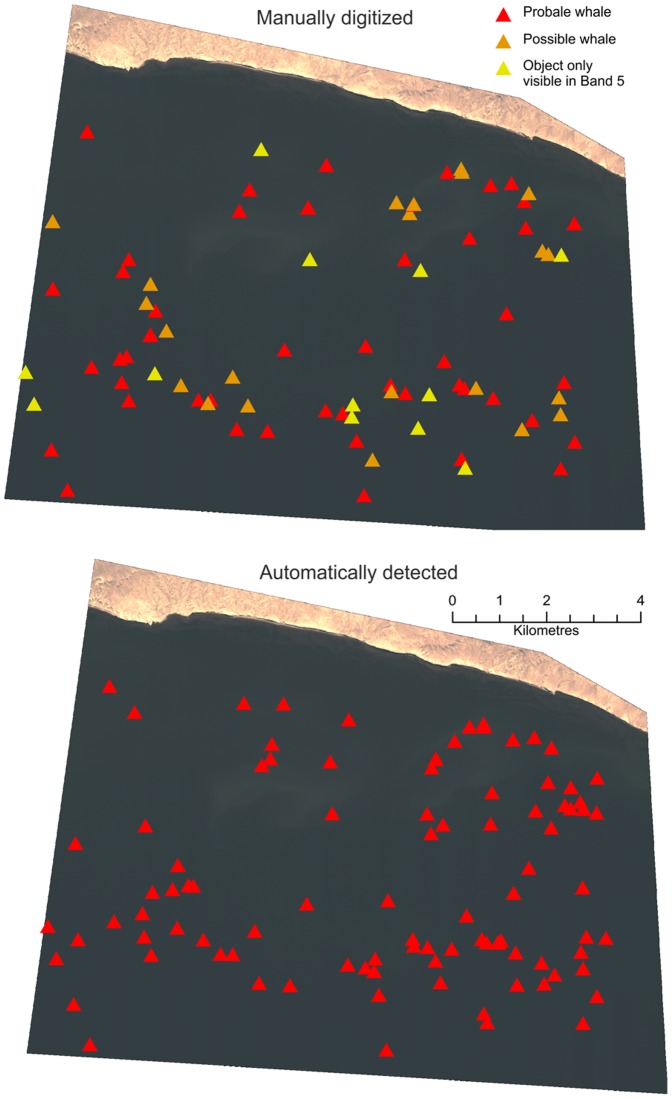
Comparison between manually identified and automatically identified whales. Manually identified whales (top) have been broken into three classes; shapes that are whale-like and whale-sized are classed as probable whales, other objects are classed as possible whales, but may include bubble slicks and some groups of seabirds. The third class are objects identified only in the water penetrating coastal band, these are interpreted as sub-surface feature that are potentially whales. The bottom image shows the whale-like objects identified from the thresholding analysis of the coastal band.

Returns from the four automatic analysis routines were assessed against the manually digitized data (figure 5). In this analysis we assume that manual detection detects all whales visible on the surface. Results from the supervised maximum likelihood classification returned many errors of commission in comparison to the unsupervised classification. The supervised classification also has the disadvantage of needing the input of user-derived signatures which take additional effort and inserts user bias into any classification. No meaningful results could be obtained using this method. The two unsupervised classification methods gave reasonable results, but the results that best matched the manually counted data came from a simple thresholding of single bands (table 1). The two most effective bands were the panchromatic and the water penetrating Band 5, which slightly outperformed the more detailed panchromatic analysis (see table 1). The single best routine was thresholding of the coastal Band 5; this technique found 84.6% of all manually digitized whales and 89% of the objects manually classed as probable whales, with 23.7% false positives. However, thresholding also requires user input to identify thresholds and therefore the greater accuracy of the technique needs to be balanced against the need for extra manual input.

**Table 1:** Assessment of results from four automatic detection techniques in relation to manually digitized whales; results from two unsupervised classification techniques and two Thresholding analyses.

	Manually digitized		Unsupervied iso means	Unsupervised kmeans	Threshold Panchromatic	Threshold Band 5
total signals	91	total signals	158	102	64	101
probable	55	probable matches	44	42	43	49
possible	23	possible matches	16	11	14	15
Band 5 only	13	band 5 matches	1	0	0	13
		total found	61	53	57	77
		% found	67.0	58.2	62.6	84.6
		% of probable	80.0	76.4	78.2	89.1
		total missed	30	38	34	14
		% missed	33.0	41.8	37.4	15.4
		false positives	97	49	7	24
		% false positives	61.4	48.0	10.9	23.8
		% good	38.6	52.0	89.1	76.2

## Discussion

### How do we know it is a Southern right whale?

There are many objects in the image that resemble whales, but the question remains; how do we know it is a Southern right whale? The answer to this can be broken into three criteria used to identify any objects in remotely sensed imagery:

1. The object is the right size and shape to be a whale
2. The object is in a place we would expect to find whales
3. There are no (or few) other types of objects that could be misclassified as whales to cause errors of commission.

In this study we have digitized and automatically identified objects that are the right size (up to 16 m long) and shape. Although the size of the whales has an upper limit the lower limit is difficult to assess as the deeper the whale in the water column the less we are likely to see. The shape is generally ellipsoidal, although this can vary due to rolling, tail slapping and bubbles and other ripples associated with the animal. In the location of the study at the time the image was taken we expect to see a high density of whales in the image, especially mothers which, at this time of year, are forced to swim at the surface to support their calves.

There are only a limited number of other confounding artefacts that could cause errors of commission: No other large marine mammals are reported to frequent this bay, right whales are the only large whale species that regularly use the shallow calving grounds of Peninsula Valdés [24]. Orcas, much smaller in size, are common in the area, although at a different time of the year, and are unlikely to be confused with right whales. This is an important criteria for the study area as it seems unlikely that different baleen species could be differentiated with the resolution of currently available satellite data. Of the other possible confounding factors the most likely are subsurface rocks in very shallow areas, seabird groups, surface bubbles and boats. Surface bubbles and seabird groups may include whales beneath them but it is unlikely that a single image of this resolution can elucidate whether a whale exists within these features. Therefore, when digitizing we classed whale-like features as “probable whales” and weaker signals, that may include seabird groups and surface bubbles, as “possible whales”. Some of these issues, such as discrimination between whales and subsurface rocks, could be resolved with the purchase of multiple imagery or stereo-pairs where movement of whales between images would eliminate the possibility of rocks awash or at the surface. Boats should be identifiable by their uniform pale colouration, wakes or strong outlines which discriminate them visually from the typical signatures of whales. In the previous Abileah study using lower resolution imagery stationary boats could be clearly identified [17]. The WorldView2 imagery used in our study has 2.5 times as many pixels per unit area as the IKONOS data and we would therefore expect that boats either stationary or moving could be discriminated from whales in the manual search. In the section of Golfo Nuevo contained in our image no such features were identified.

**Figure 6:**
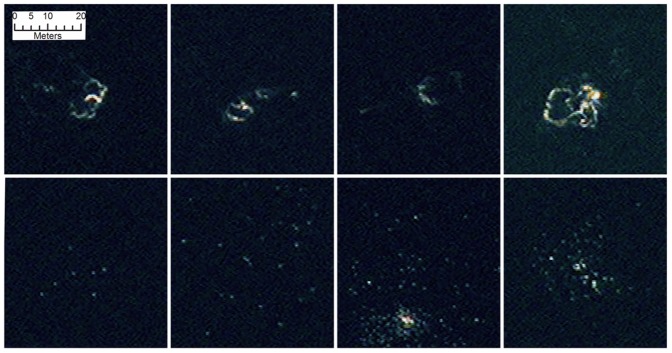
Examples of possible confounding features found in the image (false colour bands 1-8-5). The top row shows examples of surface features that are probably bubbles from subsurface whales. Whether the whales are still under the bubble areas is difficult to ascertain. The lower row show clusters white dots, probably seabirds. Seabirds have been recorded to feed on whales at Península Valdés (see discussion). The third (and possible fourth) of these images shows a larger white object that could be a whale (or whale carcass) although once more it is impossible to tell with any certainty in this imagery. Reprinted under a CC BY license with permission from British Antarctic Survey and DigitalGlobe.

On several potential whale objects there is a strong return at one end of the feature which is likely to be from calluses on the whale’s head, a feature which could aid automatic detection. Several objects identified as whales could be interpreted as pairs, or as a mother and calf, others may be displaying behaviour such as tail slapping, rolling or blowing (see figure 6). These behaviours present challenges for automatic analyses.

The results from the automated analysis suggest that a thresholding of the water penetrating Band5 returns the best results, finding 89% of features classed as probable whales in the manual count. Thresholding a single band is a very simple technique, although it does require some user input to identify the best thresholds. The greater accuracy of the technique (in relation to the more automated unsupervised analysis) needs to be balanced against the need for extra manual input in relation to other methods. These results however are promising and suggested that larger surveys over whole calving areas, which could potentially measure thousands of square kilometres, could be automated with a degree of success using these techniques.

### Challenges and Future Improvements

The next challenge is to determine detection probabilities and understand whether counts from images can be used as a reliable index for population size, or presence. This paper shows that automated analysis of satellite imagery can achieve a good match with manual counts, but more work is needed to ensure that these manual counts are commensurate with the real number of surface whales. Once an estimate of visible whales has been formulated the ratio of visible whales versus invisible whales (those at depth or not at the breeding locality) is required to ascertain the total population size. One critical factor is estimating how deep the satellite sensor is seeing into the water column; the greater the penetration, the larger the proportion of the total population that will be identified. Penetration varies with water turbidity and surface roughness, two factors that may change over short time-spans and spatially within the image. Some estimation of turbidity may be made by comparison of the infra-red bands to the visible bands [25] although this would not account for scattering due to surface roughness. To give some true indication of water column penetration we suggest that any larger study should have submarine reflectance panels placed at depths of 5 m to 30 m large enough to be seen in the satellite image and to enable a pragmatic estimation of the depth at which whales may be visible. We have compared a number of automated techniques that will aid the up scaling of similar studies, an important consideration if remote sensing of whales using VHR optical imagery is to be expanded to cover larger areas. Our studies have concentrated on pixel based analysis [26], but object-orientated analysis or textual analysis [27], [28] may also provide comparable results (although confounding behaviours such as rolling, bubble blowing and associated surface waves may make this approach difficult).

The behaviour of right whales, with mothers calving in very shallow waters in protected bays, makes them an ideal candidate for the automated analysis of satellite imagery. The right whale population at Peninsula Valdés was previously thought to be recovering well, but recent years have seen persistent events of calf mass mortalities, suggesting major changes which require re-assessment; the latest available population estimates are over a decade old [12]. In addition, satellite image analysis offers the opportunity to repeatedly assess the number of dead whale calves washed up on beaches and even those at sea which are separated from their mothers. An additional recent threat to the southern right whale population at Peninsula Valdés has been predation by seabirds that peck blubber from calves which, when young, stay near the surface [29]. Given the potential for the WorldView2 images to identify sea bird assemblages in relation to whales it may be possible to use them to monitor, and even quantify to some degree, occurrences of this behaviour (figure. 6).

## Conclusions

We have shown that the use of current satellite imagery can be used to identify individual whales both at, and just below, the surface. The methods described here readily lend themselves to the calculation of population abundance estimates and suggest that behavioural patterns could also be elucidated. The automation of the methods means that counts can be carried out more quickly and efficiently than using traditional methods. This will allow a greater frequency of counts, both within and between years, that should lead to more robust population estimates, and the build up of a time series to asses trends. The important differences between our approach and a previous relatively unsuccessful attempt to identify whales from satellite imagery are the improvements in the on-the-ground resolution of panchromatic imagery and the use of the costal band (band 5) that penetrates to subsurface whales. These improvements allow a reasonable confidence to be assigned to the identification of individual whales thus allowing counts of whales in the wild as opposed to observations of animals in captive tanks.

A working system of whale population assessment by remote sensing will be an important new method that is potentially applicable other species of whale. Many species of whale breed in areas of calm water where, in order to protect their vulnerable calves, females remain close to the surface e.g. Humpbacks (*Megaptera novaeangliae*). Such behaviours would allow the methods outlined here to be used for population estimates.

Our methods can potentially help providing within and between season population estimates, changes in distribution and use of the breeding grounds, both for right whales and other species of whale that breed in sheltered locations. Importantly, future satellite platforms planned in 2013 and 2014 will increase the on-the-ground resolution of panchromatic imagery from ∼50 cm to 34 cm and coastal band from ∼2 m to 1.24 m (Worldview3 planned launch 2014). This will result significantly higher quality imagery and therefore, greater confidence in identifying whales and differentiating mother calf pairs. Such improvements will also provide the opportunity to expand similar methodologies to other whale species.
